# Expression of Hox genes during regeneration of nereid polychaete *Alitta (Nereis) virens* (Annelida, Lophotrochozoa)

**DOI:** 10.1186/2041-9139-4-14

**Published:** 2013-05-02

**Authors:** Elena L Novikova, Nadezhda I Bakalenko, Alexander Y Nesterenko, Milana A Kulakova

**Affiliations:** 1 Department of Embryology, Laboratory of Experimental Embryology, Saint-Petersburg State University, Oranienbaumskoe sh., 2, Petergof, Saint Petersburg, Russia

**Keywords:** Regeneration, ncRNA, Hox genes, Positional information, Polychaetes, Lophotrochozoa

## Abstract

**Background:**

Hox genes are the key determinants of different morphogenetic events in all bilaterian animals. These genes are probably responsible for the maintenance of regenerative capacities by providing positional information in the regenerating animal body. Polychaetes are well known for their ability to regenerate the posterior as well as the anterior part of the body. We have recently described the expression of 10 out of 11 Hox genes during postlarval growth of *Alitta (Nereis) virens*. Hox genes form gradient overlapping expression patterns, which probably do not contribute to the morphological diversity of segments along the anterior-posterior axis of the homonomously segmented worm. We suggest that this gradient expression of Hox genes establishes positional information along the body that can be used to maintain coordinated growth and regeneration.

**Results:**

We showed that most of the Hox gene expression patterns are reorganized in the central nervous system, segmental ectoderm and mesoderm. The reorganization takes place long before regeneration becomes apparent. The most rapid reorganization was observed for the genes with the largest differences in expression levels in the amputation site and the terminal structures (pygidium and growth zone). Moreover, we revealed the expression of two antisense Hox RNAs (*Nvi-antiHox5* and *Nvi-antiHox7*) demonstrating unique expression patterns during regeneration.

**Conclusions:**

Hox genes probably participate in the maintenance and restoration of the positional information in *A. virens*. During postlarval growth and regeneration, Hox genes do not alter the diversity of segments but provide the positional information along the anterior-posterior axis. The reorganization of at least some Hox gene patterns during regeneration may be regulated by their anti-sense transcripts, providing a rapid response of Hox gene transcripts to positional failure. The capacity of Hox genes to maintain the positional information in the adult body is present in different bilaterian animals (planarias, polychaetes and mammals) and might be an ancestral function inherited from the common evolutionary remote ancestor.

## Background

Regeneration capacity stands for the ability of the adult organism to restore lost parts of the body by *de novo* growth due to cell proliferation and/or reorganization of somatic tissues [1]. Regeneration processes are studied in many animal models, including anamniotes (Anura and Urodella, fishes), insects (cricket), planaria and hydrozoa (*Hydra*), and mammals [2-6]. Intriguingly, regenerative capacities are dramatically different in different animals. On the one hand, planarians can grow any part of the body and even regenerate completely from a tiny body fragment, consisting of 1.5–4 thousands of cells, which makes up about 6–10% of the total cell count [7]. On the other hand, mammals are capable of physiological regeneration [8-10] and can restore parts of internal organs after injury, e.g., the heart after myocardial infarction or the bone after fracture [11,12]. Newborn mice and children can regenerate fingers after injury or amputation of the distal phalanx [5].

It was noticed years ago that molecular programs of reparative and embryological morphogenesis have several features in common. For instance, embryonic patterns of key transcriptional factors are restored during regenerative processes, e.g., in the case of Sonic hedgehog (*Shh*), *FGF*, *Wnt* and Hox genes [3,13-16]. The question arises what differences in molecular factor functioning during regeneration of different animals define their varying capacities for reparative morphogenesis.

Hox genes encode transcriptional factors, which are involved in multiple morphogenetic processes [17]. Their main and conservative function is considered to be the regionalization of the anterior-posterior (AP) axis in both protostome and deuterostome animals during embryonic development. Hox genes pattern the bilateral body according to the rules of spatial and temporal colinearity [18]. At the same time, Hox genes were also shown to participate in processes in the adult body. Hox genes are necessary for reparative morphogenesis in different model organisms, e.g. planaria, zebrafish, axolotl and *Xenopus laevis*[4,19-22]. In higher vertebrates, mostly mammals, Hox genes are expressed in the adult tissues capable of remodeling and/or constant renewal. Thus, in vertebrates the genes of the Hox cluster are involved in physiological regeneration, e.g., cyclic renewal of hair follicles [8] or hematopoiesis [9,10]. Genes *HOXA7*, *HOXB3*, *HOXA3* and *HOXB13* were shown to regulate differentiation of mesenchymal stem cells (MSCs), which play an active role in reparative morphogenesis in vertebrates [23].

Expression analysis of genes in human dermal fibroblasts revealed differential Hox gene expression in fibroblasts with different localization. This “Hox code” is maintained during the whole cell life and is probably needed for the correct establishment of regenerative processes [24]. It is becoming obvious that one of the main Hox gene functions in the adult organism is the maintenance of the positional information, which is provided by Hox proteins [25].

Previously we described the expression of 11 Hox genes in the ontogenesis of errant marine polychaetes *A. virens* (Nereididae, Annelida, Lophotrochozoa) and *Platynereis dumerilii*[26]. The ontogenesis of nereididae polychaetes comprises the stages of spherical trochophore larva, segmented nechtochaete larva and postlarval growth [26]. Hox genes demonstrated canonical collinear expression in segment larval ectoderm. However, during the formation of the definitive multisegmental worm’s body, they pattern the morphogenetic territory in a different, and unique, way. Hox genes are expressed as a gradient, their expression domains overlapping and not retaining the anterior boundaries in the postlarval segments (Bakalenko, Novikova and Kulakova, unpublished data). We suggest that in this case, Hox genes are involved in the establishment and maintenance of positional coordinates in the growing homonomously segmented body rather than in specification of segments with similar morphology. If it is indeed the case, the expression pattern of Hox genes should be reorganized after positional failure, for example, due to a loss of a body part.

Here we studied the expression dynamics of Hox genes during different regeneration stages of the polychaete *A. virens*. Nereid polychaetes, capable of rapid unipolar regeneration, are an excellent model for studying reparative morphogenesis. We believe that characteristic features of Hox gene behavior during regeneration of *A. virens* will support our hypothesis concerning the role of these genes in creating the postlarval worm’s body and partially explain the capability of these polychaetes for rapid axial regeneration.

## Methods

### Animals

Adult *A. virens* were collected near “Kartesh” Marine Biological Station of the Zoological Institute (Russian Academy of Sciences) in the Chupa Inlet of the White Sea. Mature worms were caught with a hand net near the water surface during their spawning period (June and July). Artificial fertilization and cultivation of the embryos were carried out at 10.5°C [27]. The culture of postlarval animals was kept in the laboratory of experimental embryology (Petergof, Russia) under the following conditions: temperature, 18°C; salinity, 23^0^/_00_; artificial sea water (Red Sea salt).

### Cloning of *A. virens* Hox genes

*A. virens* Hox genes were cloned as described previously [26]. Gene fragments, except *Nvi-Hox3*, were inserted into pGEM®-T Easy Vector (Promega). *Nvi-Hox3* was inserted into pBluescript II SK^+^ (Fermentas). The vector sequence allows one to obtain sense and antisense probes from different promoters (T7 and Sp6). Antisense probes were used for the detection of sense transcripts’ expression. Sense probes were used for antisense transcripts’ detection.

### Experimental conditions

Juvenile worms consisting of 20–30 segments were relaxed in clove oil (Sigma) with a low concentration for 5 min and then cut into two pieces approximately in the middle of the body. The anterior parts and the “tails” were incubated separately, each part in a separate petri dish (3 cm in diameter). Regenerating worms were fixed with 4% PFA in 1.75× PBS at the following time points: 0 h, 4 h, 10 h, 18 h, 1, 2, 3 and 7 days. Eight to ten worms were used for each time point in *in situ* hybridization.

### Whole mount *in situ* hybridization (WMISH)

WMISH was performed for *A. virens* as described in Irvine et al. (1999) [28] with some modifications. A detailed protocol is available upon request. Digoxigenin-labeled RNA probes were prepared according to the manufacturer’s protocol (Roche). Collagenase treatment [collagenase (Sigma) 100 γ/ml, 2.5 mM DTT; 1mM CaCl_2_] was used for 5 min, and incubation in SDS/Tween buffer was performed for 30 min to improve probe penetration. Proteinase K (Sigma) treatment was performed for 8–10 min (10 γ/ml). Prehybridization and hybridization steps in Hybridization (Hybe) buffer were carried out overnight at 65°C. Washings from the probe were performed as follows: 100% Hybe 2 × 60 min, 80% prehybe/20% PTw 2 × 20 min, 50% prehybe/50% PTw 4 × 30 min, 20% prehybe/80% PTw 2 × 20 min and 100% PTw 2 × 20 min at 67°C. Incubation in the blocking buffer [1% B-M Blocking Rgt. (Roche)/5% normal sheep serum/PBS/0.1% Tween] took 60 min. Incubation in Anti-Digoxigenin-AP, Fab fragments from sheep (Roche) (1:4,000) was performed overnight at +4°C. Washings from antibodies were carried out for 10 × 20 min in PTw on the shaker. Incubation in AP buffer was performed before colored reaction for 3 × 5 min. BM-purple (Roche) was used as a chromogenic substrate to localize the hybridized probe. The results were imaged on a DMRXA microscope (Leica) with a Leica DC500 digital camera with Nomarski optics. The worms were mounted in clove oil before the microscopic analysis. Optical sections were assembled with the use of Helicon Focus software. Brightness, contrast and color values were corrected in all the images using image processing software Adobe Photoshop CS5. SDS/Tween buffer: 150 mM NaCl, 50 mM Tris, pH 7.5, 1 mM EDTA, 1% SDS, 0.5% Tween 20. Hybridization buffer: 50% formamide, 5× SSC pH 4.5, 50 μg/ml yeast tRNA, 50 μg/ml heparin, 0.1% Tween 20, 1% SDS, 100 μg/ml salmon ssDNA. Prehybe buffer: 50% formamide, 5× SSC pH 4.5, 1% SDS, 0.1% Tween 20. PBS: 1.5 M NaCl, 70 mM Na_2_HPO_4_, 30 mM NaH_2_PO_4._ PTw: PBS + 0.2% Tween 20. AP buffer: 100 mM NaCl, 50 mM MgCl_2_, 100 mM Tris, pH 9.5, 0.01% Tween 20. *In situ* hybridization for each time point was performed for at least 3 times.

## Results

### Regeneration time course

Though many polychaetes are capable of extensive regeneration, members of the Nereidae can restore only the posterior body end. During posterior regeneration the pygidial structures and prepygidial growth zone (GZ) are formed first, and the segments form after that sequentially, as during normal growth. By 0–4 h post-amputation (hpa), the edges of the wound are tightened as a result of ring muscle contraction, and the gut lumen is closed. The gut comes in tight contact with the ectoderm to prevent the efflux of fluid from the coelomic cavity. The blastema is formed around 1 day post amputation (dpa). The cells of the superficial epithelium around the wound proliferate and migrate toward the injury. By 2 dpa, two rudiments, the primordia of the pygidial lobes, appear on the ventral side laterally in respect to the anus. The pygidium of the regenerating worm, with two well-developed lobes and anal cirri, is fully formed after 3 days. Neither parapodial rudiments nor any signs of segmentation can be seen on the surface of the regenerating region. However, the beginning of the segmentation process can be visualized on histological sections (Starunov and Lavrova, personal communication). After the pygidium and the GZ have been formed, the segmentation process proceeds as during normal growth.

### Hox gene expression during the regeneration of *A. virens*

We divided Hox genes into four groups by their expression dynamics during the posterior regeneration of *A. virens*.

### Early response genes

The first group comprises *Nvi-Lox5*, *Nvi-Lox2* and *Nvi-Post2*. The expression patterns of these genes are reorganized very early in the neural system, within 4 h after amputation.

#### Nvi-Lox5

**Figure 1:**
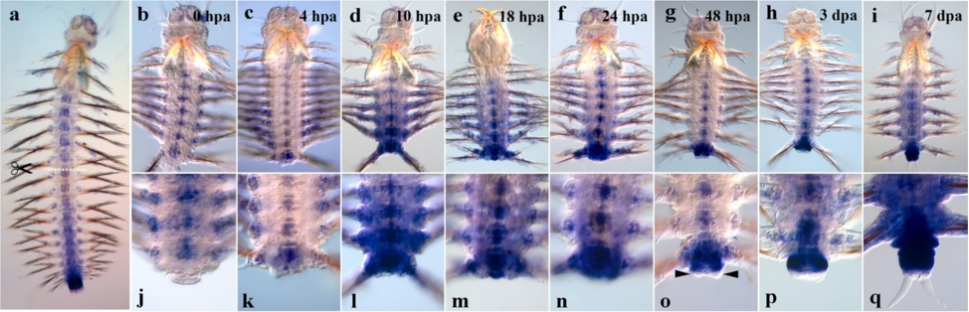
**Expression of *Nvi-Lox5 *during postlarval development (a) and regeneration (b–q).** Anterior is directed upwards on all panels. All views are ventral. (**a**) Expression pattern of *Nvi-Lox5* in juvenile worm during normal growth. Expression forms posterior-anterior gradient and covers the GZ. Expression of *Nvi-Lox5* at 0 hpa (**b**, **j**), 4 hpa (**c**, **k**), 10 hpa (**d**, **l**), 18 hpa (**e**, **m**), 24 hpa (**f**, **n**), 48 hpa (**g**, **o**), 3 dpa (**h**, **p**) and 7 dpa (**i**, **q**). *Black arrowheads* mark *Nvi-Lox5*-negative forming pygidial lobes (**o**). Magnification 20×. For details, see text.

*Nvi-Lox5* has a high expression level in the middle of the body during normal growth (Figure 1a), so the gene transcription is easily detected at the amputation site immediately after the operation (Figure 1b, j). The anterior expression border lies in the second chaeta-bearing (third larval) segment, persisting there throughout the regeneration process. Intensive gene expression is visible in the neural cord and segmental ectoderm by 4 hpa not only in the last body segment, but also in several previous ones (Figure 1c, k). By 18 hpa, the *Nvi-Lox5* expression domain in the segmental ectoderm narrows down, and the expression maximum is detected in the last body segment (Figure 1e, m). By 2 dpa, the expression domain in the segmental ectoderm is restricted to the segment closest to the amputation site and the forming GZ (Figure 1g, o). Pygidial lobe buds are *Nvi-Lox5*-negative (Figure 1o, black arrowheads). The expression level in the nerve cord is at the same level or slightly lower than at the previous stage. By 3 dpa, the gene transcription persists in the neural system, the last body segment and the forming GZ (Figure 1h, p). In a week-old regenerating worm, the *Nvi-Lox5* pattern characteristic of normal growth is mostly restored. The transcript is detected in the ventral neural cord (VNC), the segmental ectoderm and GZ (Figure 1i, q).

#### Nvi-Lox2

**Figure 2:**
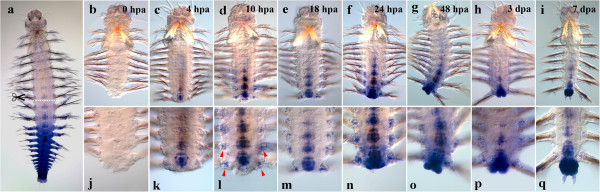
**Expression of *Nvi-Lox2 *during postlarval development (a) and regeneration (b–q).** Anterior is directed upwards on all panels. All views are ventral. (**a**) Expression pattern of *Nvi-Lox2* in juvenile worm during normal growth. Expression forms posterior-anterior gradient and covers the GZ. Expression of *Nvi-Lox2* at 0 hpa (**b**, **j**), 4 hpa (**c**, **k**), 10 hpa (**d**, **l**), 18 hpa (**e**, **m**), 24 hpa (**f**, **n**), 48 hpa (**g**, **o**), 3 dpa (**h**, **p**) and 7 dpa (**i**, **q**). *Red arrowheads* mark *Nvi-Lox2*-positive domains at the base of parapodia (**l**). Magnification 20×. For details, see text.

The level of *Nvi-Lox2* transcription is undetectable at the amputation site immediately after the operation (Figure 2b, j). Nevertheless, by 4 hpa, the gene expression reaches a high level in the ganglia of the two old segments at the amputation site (Figure 2c, k). As a result, a short posterior-anterior gradient is restored in the nerve cord. By 10 hpa, the neural expression moves toward the head, a couple of ganglia more thus becoming *Nvi-Lox2* positive (Figure 2d). The expression domains appear at the base of the parapodia (Figure 2l, *red arrowheads*). The gene transcription is activated in superficial and deep tissues in the last old segment of the body by 1 dpa (Figure 2n). The anterior expression boundary in the neural system shifts toward the head, and the expression is detected in about half of the body ganglia (Figure 2f). The gradient expression pattern is maintained at the base of parapodia. By 2 dpa, a strong expression is visible in the regenerative bud. The gradient expression in the nerve cord remains mostly unchanged. A weak transcription is detected now in the segmental ectoderm of several last body segments (Figure 2g, o). By 3 dpa, an intensive expression is visible in the formed pygidium and the GZ (Figure 2p). The gradient mode of the expression is retained in VNC ganglia, though the gradient becomes much shorter as the anterior expression domain moves backwards (Figure 2h). The expression domains in the base of parapodia persist, too. The nascent segments display a very high transcription level of *Nvi-Lox2* by 7 dpa. The anterior expression boundary is diffused, being retained in 6–8 chaeta-bearing segments (Figure 2h, q).

#### Nvi-Post2

**Figure 3:**
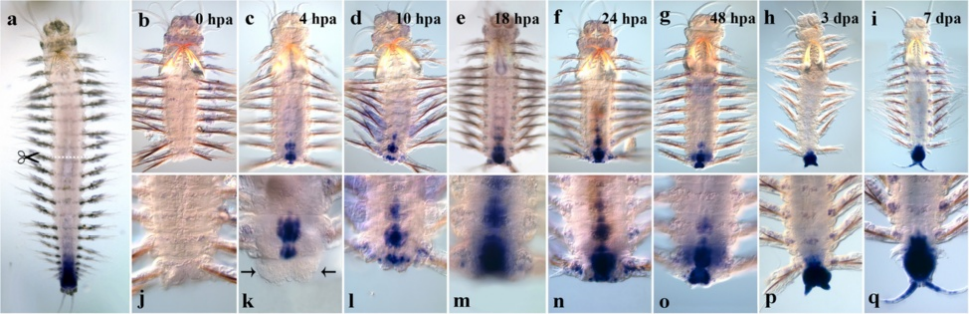
**Expression of *Nvi-Post2 *during postlarval development (a) and regeneration (b–q).** Anterior is directed upwards on all panels. All views are ventral. (**a**) Expression pattern of *Nvi-Post2* in juvenile worm during normal growth. Expression forms posterior-anterior gradient and covers the GZ. Expression of *Nvi-Post2* at 0 hpa (**b**, **j**), 4 hpa (**c**, **k**), 10 hpa (**d**, **l**), 18 hpa (**e**, **m**), 24 hpa (**f**, **n**), 48 hpa (**g**, **o**), 3 dpa (**h**, **p**) and 7 dpa (**i**, **q**). *Black arrows* (**k**) mark protruded gut. Magnification 20×. For details, see text.

The transcription of the most posterior gene *Nvi-Post2* is also upregulated by 4 hpa in differentiated cells of the nervous system near the amputation site (Figure 3c, k). The number of *Post2*-positive ganglia reaches 3–4 by 10 hpa (Figure 3d, l). The transcription intensifies in the last segment by 1 dpa, the neural system of other segments retaining the same expression level (Figure 3f, n). A slight downregulation of the expression takes place by 48 hpa. The transcription spreads to the inner cells of the regenerative bud, but its epithelium is *Post2*-negative (Figure 3g, o). The apparent downregulation of the gene expression in VNC occurs by 3 dpa, the expression domain being restricted to the last body segment (Figure 3h, p). At the same time, the gene starts to be intensively transcribed in the pygidial mesoderm, cirri and formed hindgut. The pygidial ectodermal expression is barely detected. By 7 dpa, the expression in the mesoderm and the neural ganglia of the nascent segments is visible (Figure 3i, q) as well as in the pygidium and the anal cirri. The neural expression is restricted to the segment closest to the amputation site and to the ganglia of the nascent segments.

### Middle response genes, expressed in neural system

*Nvi-Hox5* and *Nvi-Hox7* display pattern reorganization by 10 and 18 hpa, respectively, before the active proliferation starts.

#### Nvi-Hox5

**Figure 4:**
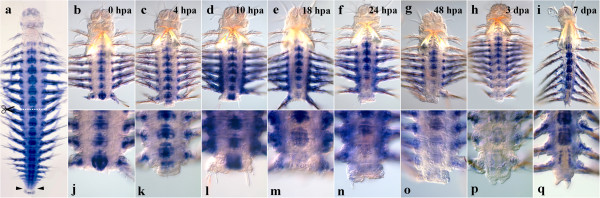
**Expression of *Nvi-Hox5 *during postlarval development (a) and regeneration (b–q).** Anterior is directed upwards on all panels. All views are ventral. (**a**) Expression pattern of *Nvi-Hox5* in juvenile worm during normal growth. Expression forms anterior-posterior gradient and does not spread to the GZ (*black arrowheads*). Expression of *Nvi-Hox5* at 0 hpa (**b**, **j**), 4 hpa (**c**, **k**), 10 hpa (**d**, **l**), 18 hpa (**e**, **m**), 24 hpa (**f**, **n**), 48 hpa (**g**, **o**), 3 dpa (**h**, **p**) and 7 dpa (**i**, **q**). Downregulation of gene transcription at the amputation site starts at 10 hpa (**d**, **l**). Magnification 20×. For details, see text.

Immediately after the operation, the *Nvi-Hox5* expression maximum is at the amputation site (Figure 4j). The gene transcript is detected in the VNC ganglia, segmental ectoderm and parapodia from the second chaeta-bearing (third larval) segment to the amputation site (Figure 4b, j). The expression pattern generally remains unchanged by 4 hpa (Figure 4c, k). A remarkable local reorganization of the *Nvi-Hox5* expression pattern occurs by 10 hpa. The expression level decreases significantly in the last body segment, mostly in the segmental ectoderm (Figure 4d, l). The expression level of *Nvi-Hox5* in the ganglia of the last two segments decreases by 18 hpa (Figure 4e, m). After 1 day of regeneration, downregulation of neural expression is visible in two or three of the last body segments (Figure 4f, n). By 2 dpa, the posterior boundary of *Nvi-Hox5* expression moves even further to the head. It is clear that the *Hox5*-transcript disappears not only from the neural tissue, but also from the parapodia and the segmental ectoderm (Figure 4g, o). The pygidial zone of a 3-day-old regenerating worm is *Hox5*-negative (Figure 4p). Interestingly, the transcript can be detected in the last old body segment again a week after the operation, when new segments are formed (Figure 4i). It is also noteworthy that in the ganglion of the last adult segment, the expression is detected only in the lateral neurons, but not in those on the midline (Figure 4q). The anterior boundary persists in the second chaeta-bearing segment throughout the regeneration process.

#### Nvi-Hox7

**Figure 5:**
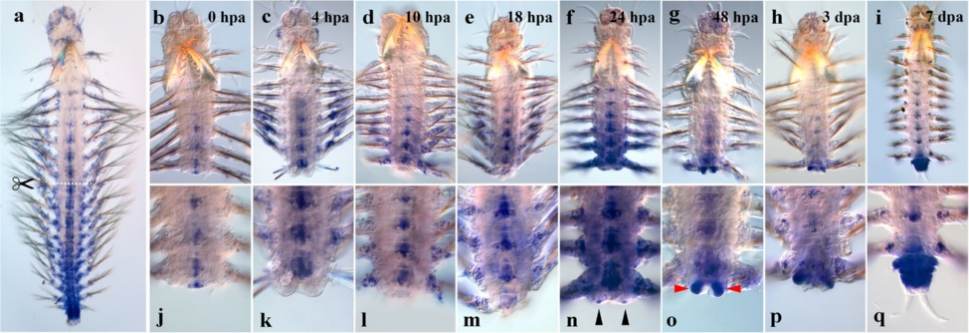
**Expression of *Nvi-Hox7 *during postlarval development (a) and regeneration (b–q).** Anterior is directed upwards on all panels. All views are ventral. (**a**) Expression pattern of *Nvi-Hox7* in juvenile worm during normal growth. Expression forms posterior-anterior gradient and covers the GZ. Expression of *Nvi-Hox7* at 0 hpa (**b**, **j**), 4 hpa (**c**, **k**), 10 hpa (**d**, **l**), 18 hpa (**e**, **m**), 24 hpa (**f**, **n**), 48 hpa (**g**, **o**), 3 dpa (**h**, **p**) and 7 dpa (**i**, **q**). *Black arrowheads* mark *Hox7*-positive cells under wound epithelium (**n**). *Red arrowheads* mark prominent expression in the nascent GZ (**o**). Magnification 20×. For details, see text.

During normal growth, *Nvi-Hox7* is expressed in a vast posterior-anterior gradient (Figure 5a). The transcript is detected at the amputation site, and its expression is visible in the ganglia of VNC immediately after amputation (Figure 5b, j). This expression domain does not retain a fixed anterior boundary, which is detected in segments 5–7 in different worms regardless of their size and age. The expression level gradually increases in the ganglia from 4 to 10 hpa (Figure 5c, d). A weak ectodermal expression is visible in a number of segments at the posterior end of the body (Figure 5k, l). Apart from that, the pattern remains unchanged. The transcription level in the last segment before the amputation site slightly decreases by 18 hpa (Figure 5e, m). By 24 hpa, the expression level generally increases, but the ectoderm at the amputation site remains *Nvi-Hox7*-negative (Figure 5f, n). Under the wound epithelium, bilateral patches of *Hox7*-positive cells appear (Figure 5n, black arrowheads). In some worms the anterior expression boundary moves toward the larval territory (two first setae-bearing segments), but never crosses the border between the larval and the postlarval segments. By 48 hpa, the expression level in VNC decreases significantly, a strong signal being nevertheless maintained in the ganglion of the last adult segment (Figure 5g). A prominent expression is detected in the nascent GZ (Figure 5o, red arrowheads). In 3 dpa, the expression pattern remains unchanged. A strong expression domain marks the formed prepygidial GZ and the bud of the nascent segment. Pygidial lobes are *Nvi-Hox7*-negative (Figure 5h, p). In a week after amputation, the domains in ganglia still display a low expression level, but an intensive expression is detected in the entire nascent segments, including the forming parapodia (Figure 5i, q).

### Middle response genes, expressed in the growth zone

This group comprises *Nvi-Hox2* and *Nvi-Hox3* genes. Both of them do not form gradients in the postlarval body and are activated *de novo* in 10 hpa.

#### Nvi-Hox2

**Figure 6:**
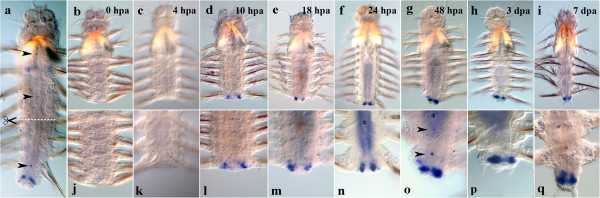
**Expression of *Nvi-Hox2 *during postlarval development (a) and regeneration (b–q).** Anterior is directed upwards on all panels. All views are ventral. (**a**) Expression pattern of *Nvi-Hox2* in juvenile worm during normal growth. Expression forms a ring in ectodermal and mesodermal tissues of the GZ. *Red arrowheads* mark single-cell expression domains in segment ectoderm. Expression of *Nvi-Hox2* at 0 hpa (**b**, **j**), 4 hpa (**c**, **k**), 10 hpa (**d**, **l**), 18 hpa (**e**, **m**), 24 hpa (**f**, **n**), 48 hpa (**g**, **o**), 3 dpa (**h**, **p**) and 7 dpa (**i**, **q**). *Black asterisks* mark background in the gut. Magnification 20×. For details, see text.

The cut passes through the part of the body with no detectable expression of *Nvi-Hox2* (Figure 6a, b, j). During postlarval development, besides the *Nvi-Hox2* expression domain in GZ, there are also single *Nvi-Hox2*-positive cells on the midline of every ganglion, beginning from the first chaeta-bearing segment. The expression intensity reaches its maximum in several anterior and several posterior adult segments (Figure 6a, arrowheads). The expression is upregulated by 10 hpa in two bilateral domains at the amputation site. The transcript is detected in superficial cells as well as in cells located between the gut and the epithelium (Figure 6d, l). The expression becomes more intensive by 2 dpa, spreading to the mesodermal and the ectodermal parts of the area between the forming pygidium and the last body segment (Figure 6g, o). Notably, the expression of *Nvi-Hox2* is upregulated in the single-cell domain in the ganglia of the two last body segments (Figure 6g, o, arrowheads), which is actually characteristic of the last adult body segments during normal development. By the 7th dpa, the transcript is detected in the mesodermal component of the GZ, as well as in the mesoderm and the ectoderm of nascent segments (Figure 6i, q).

#### Nvi-Hox3

**Figure 7:**
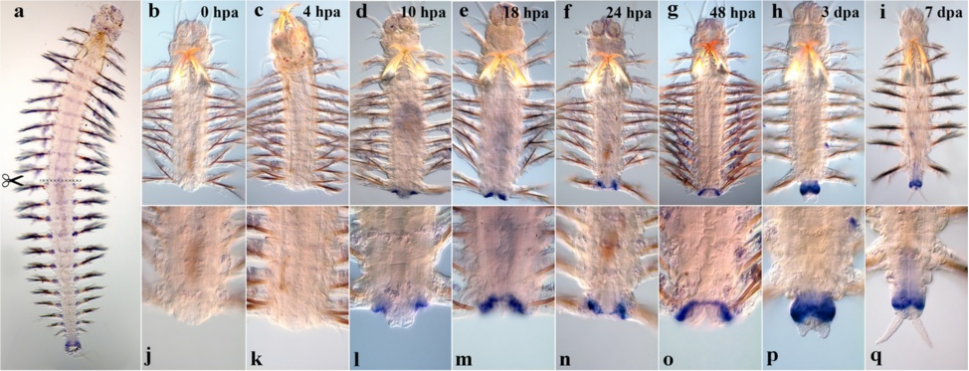
**Expression of *Nvi-Hox3 *during postlarval development (a) and regeneration (b–q).** Anterior is directed upwards on all panels. All views are ventral. (**a**) Expression pattern of *Nvi-Hox3* in juvenile worm during normal growth. Expression forms a ring in ectodermal tissues of the GZ. Expression of *Nvi-Hox3* at 0 hpa (b, j), 4 hpa (**c**, **k**), 10 hpa (**d**, **l**), 18 hpa (**e**, **m**), 24 hpa (**f**, **n**), 48 hpa (**g**, **o**), 3 dpa (**h**, **p**) and 7 dpa (**i**, **q**). Magnification 20×. For details, see text.

The first evidence of the *de novo Nvi-Hox3* expression can be seen by 10 hpa (Figure 7d, l) in the superficial domains on the border of the gut entoderm and the ectoderm, which covers the opening of the coelomic cavity at the amputation site. Eight hours later, *Nvi-Hox3*-positive cells form an irregular circumferential band opening on the dorsal and the ventral side of the worm (Figure 7e, m). The expression pattern does not change by 24 hpa (Figure 7f, n). By 2 dpa, the expression domain forms an ectodermal ring in the preblastemal area, as additional *Nvi-Hox3*-positive ectodermal cells appear (Figure 7g, o). By 3 dpa, the expression domain covers the ectoderm of the prepygidial GZ (Figure 7h, p). A week after the operation, the native expression pattern is fully restored, since in adult worms *Nvi-Hox3* transcript marks the ectodermal GZ (Figure 7i, q). Notably, the expression is restricted to this area of the *A. virens* body and does not spread to the nascent segments.

### Late response genes

The fourth group comprises the genes whose expression patterns change only with the emergence of new structures, i.e., after the start of proliferation and organogenesis.

#### Nvi-Hox1

**Figure 8:**
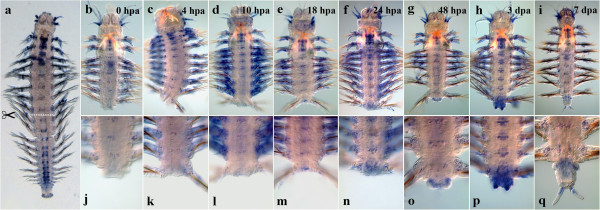
**Expression of *Nvi-Hox1 *during postlarval development (a) and regeneration (b–q).** Anterior is directed upwards on all panels. All views are ventral. (**a**) Expression pattern of *Nvi-Hox1* in juvenile worm during normal growth. Expression is detected in neural ganglia from the first larval segment, in peristomial and anal cirri. There is no expression in the GZ. Expression of *Nvi-Hox1* at 0 hpa (**b**, **j**), 4 hpa (**c**, **k**), 10 hpa (**d**, **l**), 18 hpa (**e**, **m**), 24 hpa (**f**, **n**), 48 hpa (**g**, **o**), 3 dpa (**h**, **p**) and 7 dpa (**i**, **q**). Magnification 20×. For details, see text.

In the area of the worm’s body where the cut passes, the expression of the *Nvi-Hox1* gene is minimal (Figure 8b, j). After the amputation and during the next 18 h, it does not change significantly as compared with the normal growth (Figure 8c-e, k-m). The transcript is detected in the peristomial cirri, in the anterior VNC ganglia and on the border between the pharynx and the middle gut (esophagus). The anterior expression boundary in the VNC lies in the first larval segment, which looses chaetae, merges with the head and becomes a part of the head structures of the juvenile worm. The first evidence of upregulation of *Nvi-Hox1* expression can be seen by 24 hpa (Figure 8f, n). *De novo* expression becomes visible in the ganglion, closest to the amputation site. The expression vanishes in 2 days, but is upregulated again in the rudiment of the future pygidium (Figure 8g, o). The intensive expression arises within the developing anal cirri buds (Figure 8h, p) and persists there at later stages. In the nascent segments (7 dpa), the transcript is detected in VNC ganglia. The ganglial expression pattern is similar to that of native worms (Figure 8i, q). Noticeably, the expression is also visible in lateral neurons of the last “old” ganglion (Figure 8i, q).

#### Nvi-Hox4

**Figure 9:**
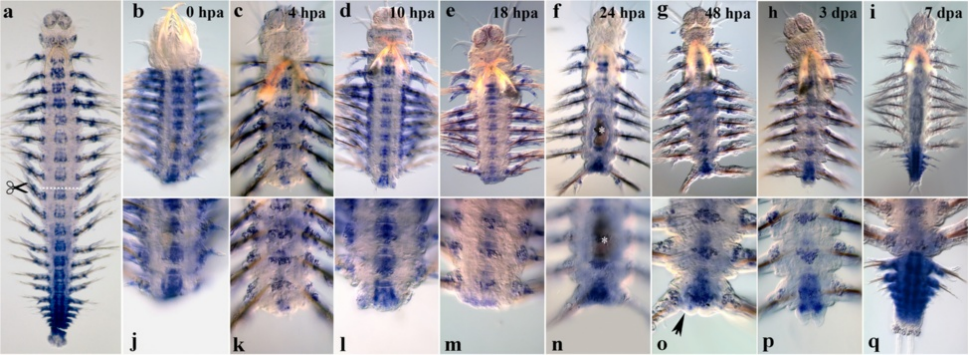
**Expression of *Nvi-Hox4 *during postlarval development (a) and regeneration (b–q).** Anterior is directed upwards on all panels. All views are ventral. (**a**) Expression pattern of *Nvi-Hox4* in juvenile worm during normal growth. The anterior boundary is stabilized in the first setae-bearing segment and expression is absent from the posterior GZ. Expression of *Nvi-Hox4* at 0 hpa (**b**, **j**), 4 hpa (**c**, **k**), 10 hpa (**d**, **l**), 18 hpa (**e**, **m**), 24 hpa (**f**, **n**), 48 hpa (**g**, **o**), 3 dpa (**h**, **p**) and 7 dpa (**i**, **q**). *Asterisks* mark food in the gut. *Black arrow* marks expression domain in mesoderm. Magnification 20×. For details, see text.

After the amputation of the posterior part of the worm (0 h), the native expression pattern is conserved (Figure 9b, j). *Nvi-Hox4* is expressed in the ganglia of ventral nerve cord with the anterior boundary in the first chaeta-bearing (the second larval) segment. The expression pattern does not generally change by 4 hpa, though the expression level seems to decrease as compared to 0 hpa (Figure 9c, k). Since *in situ* hybridization is not a quantitative method, we can determine only the spatial and the temporal pattern of gene expression, but not the exact quantitative characteristics of expression. Nevertheless, as the worms were incubated under similar experimental conditions, we can make some observations on the decrease in the expression levels comparing the different regeneration stages. The anterior boundary persists in the first postlarval segment. The expression domains at the base of the parapodia and parapodial branches are also retained. The first evidence of *Nvi-Hox4* expression upregulation is detectable by 18 hpa (but not in the neural system). A weak diffuse expression can be seen in epithelial cells covering the amputation site. The neural anterior boundary still persists in the first setae-bearing segment (Figure 9e, m). By 1 dpa, the intensity of the neural expression significantly increases in the segment closest to the amputation site (Figure 9f, n). The first signs of mesodermal expression appear in 2 dpa. The signal is detected in a number of large cells under the ectoderm of the forming pygidium (Figure 9g, o, black arrow). By 3 dpa, after the pygidium has been formed, the most prominent expression domain is detected in the ganglion of the last adult body segment. An intensive mesodermal expression marks the cells in the base of the pygidium (Figure 9h, p). The anterior expression domain in VNC persists, but loses its intensity. A week after the operation, the formation of new segments, displaying very strong *Nvi-Hox4* expression, proceeds (Figure 9i, q). The expression domain includes the neural system, the segment ectoderm and the forming parapodia. The localization of the neural anterior boundary remains the same.

#### Nvi-Lox4

**Figure 10:**
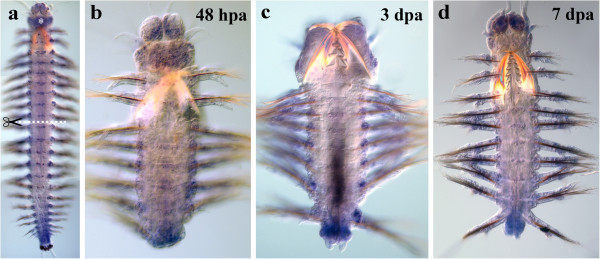
**Expression of *Nvi-Lox4 *during postlarval development (a) and regeneration (b–d).** Anterior is directed upwards on all panels. All views are ventral. (**a**) Expression pattern of *Nvi-Lox4* in juvenile worm during normal growth. Expression forms posterior-anterior gradient in VNC and mesoderm of nascent segments. Expression of *Nvi-Lox4* at 48 hpa (**b**), 3 dpa (**c**) and 7 dpa (**d**). Star marks the background (**a**). Magnification 20×. For details, see text.

*Nvi-Lox4* expression in normal growth is associated with VNC ganglia and the mesoderm of nascent segments (Figure 10a). Due to the low expression level, transcription is detected only on the following regeneration stages: 48 hpa (Figure 10b), 3 (Figure 10c) and 7 dpa (Figure 10d). By 48 hpa, the expression is detected in the ventral ectoderm of the last body segment. A weak transcription is detected in the ganglion at the amputation site and in superficial and deep tissues of regenerating parts of the body, except for the pygidium and the anal cirri (Figure 10b). However, the expression may in fact be activated earlier, and we may have failed to detect it because of a low expression level and/or a short hybridization probe (302 bp). The expression domains remain unchanged by 7 dpa (Figure 10d).

### Expression of antisense transcripts

#### Nvi-antiHox5

**Figure 11:**
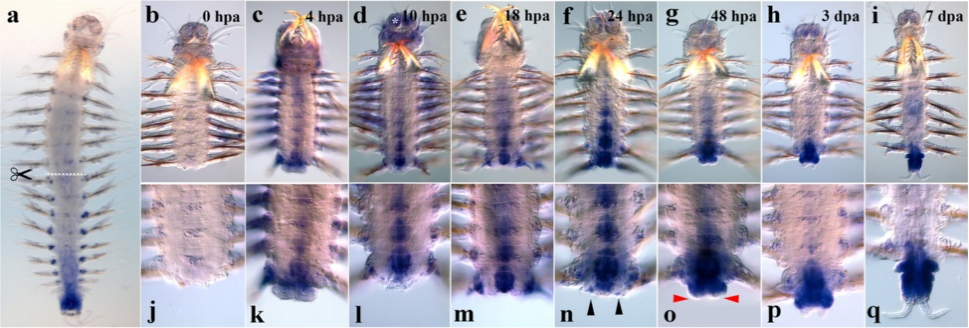
**Expression of *Nvi-antiHox5 *during postlarval development (a) and regeneration (b–q).** Anterior is directed upwards on all panels. All views are ventral. (**a**) Expression pattern of *Nvi-antiHox5* in juvenile worm during normal growth. Expression forms posterior-anterior gradient and spreads to the GZ (*black arrowheads*). Expression of *Nvi-antiHox5* at 0 hpa (**b**, **j**), 4 hpa (**c**, **k**), 10 hpa (**d**, **l**), 18 hpa (**e**, **m**), 24 hpa (**f**, **n**), 48 hpa (**g**, **o**), 3 dpa (**h**, **p**) and 7 dpa (**i**, **q**). *Black arrowheads* mark patches of large *Nvi-antiHox5*-positive cells under wound epithelium (**n**). *Red arrowheads* mark growing *Nvi-antiHox5*-negative pygidial lobes (**o**). Magnification 20×. For details, see text.

The antisense transcript of *Nvi-Hox5* (*Nvi-antiHox5*) is expressed in the last quarter of the postlarval body. No expression is detected at the amputation site immediately after the operation (Figure 11b, j). Upregulation of transcription is detectable in the segmental ectoderm and the ganglion of the last body segment by 4 hpa. This expression arises *de novo* in differentiated cells. Weak expression is also detected in the ganglia of several previous segments (Figure 11c, k). By 10 hpa, the neural expression in the last segments becomes more intensive, forming a short posterior-anterior gradient. A low-level transcription becomes detectable in patches of cells at the base of the parapodia of several last segments (Figure 11d, l). The expression domains remain unchanged by 18 hpa (Figure 11e, m). The expression becomes stronger in the segment at the amputation site by 1 dpa (Figure 11f, n). Moreover, the signal is also visible in the regeneration blastema (bilaterally symmetrical patches of large cells under the wound epithelium) (Figure 11n). By the end of the second dpa, a strong expression domain is visible in the ectodermal GZ and the underlying mesodermal tissues. The pygidial bud is *antiHox5*-negative (Figure 11o, red arrowheads). The transcript is retained in the ganglion of the last old segment, but disappears from the other segmental ganglia (Figure 11g, o). By 3 dpa, an extremely intensive expression of *Nvi-Hox5* ncRNA spreads to the GZ, underlying the mesoderm and the caudal gut. Pygidial lobes are expression-negative. A high transcription level is maintained in the ganglion of the last segment, and some signal is detected in the previous segments (Figure 11h, p). By 1 week after the operation, transcription weakens in the last old segment. It is detected in the GZ, in the mesoderm and the ectoderm of nascent segments, as well as in the gut of the newly formed part of the body (Figure 11i, q).

#### Nvi-antiHox7

**Figure 12:**
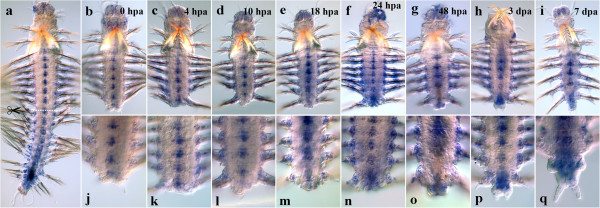
**Expression of *Nvi-antiHox7 *during postlarval development (a) and regeneration (b–q).** Anterior is directed upwards on all panels. All views are ventral. (**a**) Expression pattern of *Nvi-antiHox7* in juvenile worm during normal growth. Expression forms a posterior-anterior gradient with an anterior boundary fixed in the second to third setae-bearing segment. Expression of *Nvi-antiHox7* at 0 hpa (**b**, **j**), 4 hpa (**c**, **k**), 10 hpa (**d**, **l**), 18 hpa (**e**, **m**), 24 hpa (**f**, **n**), 48 hpa (**g**, **o**), 3 dpa (h, p) and 7 dpa (**i**, **q**). *Black arrowheads* mark expression domains in the regeneration bud (**o**). Magnification 20×. For details, see text.

Antisense transcript of *Nvi-Hox7* (*Nvi-antiHox7*) is expressed in a wide posterior-anterior gradient in VNC ganglia, at the base of parapodia and, at a low level, in the mesoderm of nascent segments. The anterior border stays in the second or third chaeta-bearing segment so that the expression does not spread to the larval territory (Figure 12a). Immediately after the amputation (0 h), the transcription is detected in the neural ganglia, the expression pattern being similar to that during the normal growth (Figure 12b, j). By 4 and 10 hpa, the pattern remains unchanged (Figure 12c, d). The pattern reorganization starts by 18 hpa: the expression is downregulated in the ganglion of the last body segment. Other expression domains do not change much (Figure 12e, m). By 24 hpa, *Nvi-antiHox7* is again upregulated in the neural ganglion and the underlying mesoderm of the last body segment (Figure 12f, n). Notably, the transcription level decreases in the neighboring segments. By 48 hpa, the transcriptional “gap” in the VNC ganglia becomes more apparent (Figure 12g, o). The transcription level increases in the regenerative bud (Figure 12o, black arrowheads). A weak ectodermal and mesodermal expression spreads to the last body segment. The rudiment of pygidial lobes is *antiHox7*-negative (Figure 12o). By 3 days after amputation dpa the expression gradient in the neural system persists. A low transcription level is detected in the second or the third chaeta-bearing segment, gradually decreasing further toward the end of the body. The expression maximum is detected in the last segment before forming the GZ and the pygidium (Figure 12h). A strong expression is visible in the mesoderm of the GZ. The pygidial area is *antiHox7*-negative (Figure 12p). A complex expression gradient in a 7-day-old regenerating worm is not so apparent (Figure 12i). A diffuse expression appears in the neuroectoderm of nascent segments and in the distal part of the formed gut. Noteworthy, the expression in the hindgut is not detected in normally growing worms (Figure 12a). It seems that more than 1 week is needed for the restoration of the native expression pattern.

## Discussion

### Hox genes in posterior regeneration of *A. virens*

Here we described the expression dynamics of 10 Hox genes during the posterior regeneration of polychaete *A. virens*. The expression was recorded at different stages of regeneration: 0, 4, 10, 18 hpa, 1, 2, 3 and 7 dpa. The 0-hpa point was taken as a control for the RNA probe penetration into the adult tissues: at this point we expected to get the same transcript distribution as in normal worms.

**Figure 13:**
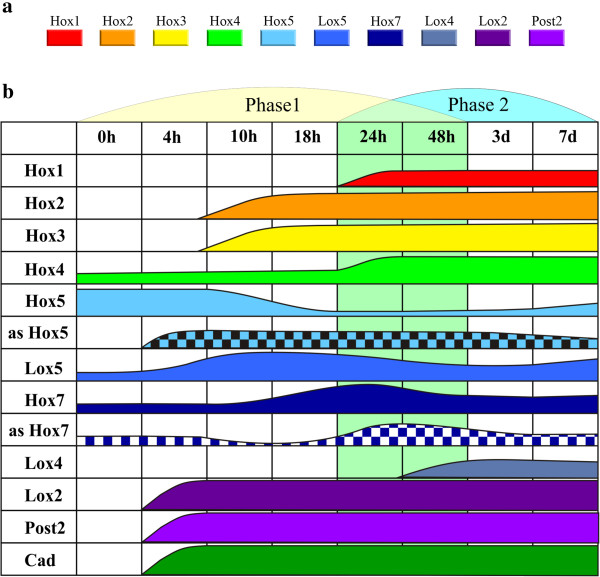
**Schematic representation of the set of *A. virens *Hox genes (a) and the expression dynamics of studied Hox genes and *Nvi-Cad *during *A. virens *regeneration (b). a**. Presumed genomic order of Hox genes in *A. virens*. **b**. Schematic representation of Hox genes’ (*different colors*), antisense transcripts’ (*checks*) and *Nvi-Cad* (*dark green*) expression on different regeneration stages of *A. virens*. The shadowing of 24 h and 48 h represent the zone of overlap of the two phases of *A. virens* regeneration. The *height of colored areas* reflects visual differences in gene expression intensity.

Based on the expression pattern of Hox genes and the morphological events, we divide the regeneration process of *A. virens* into two phases (Figure 13b). During the first phase (before 48 h), the expression patterns are reorganized and restored inside the new body boundaries. Importantly, the shift of the Hox gene expression boundaries occurs in old tissues within 18 hpa, i.e., before blastema formation. The newly established expression boundaries are maintained until the beginning of the organogenesis of the new terminal structures (pygidium and GZ).

The second regeneration phase, overlapping with the first one, starts at 24 hpa. At this stage, the blastema is formed because of proliferation of dedifferentiated cells under the wound epithelium. Most of the Hox genes are expressed intensively in the blastema from the beginning of its formation and continue to be active in the rudiment of the terminal structures, which are morphologically distinguished by 3 dpa. During this period, all the Hox genes involved in the patterning of terminal structures and nascent segments are expressed in the regenerating worm. At the onset of organogenesis, the transcription of some Hox genes is downregulated in the nervous system (Figure 2h,p; Figure 3g-i, o-q).

Recently, we have suggested a hypothesis that during postlarval growth of *A. virens,* Hox genes play an unusual role different from their function in the formation of the nectochaete body [26]. Indeed, Hox expression patterns in the larval and the postlarval body differ considerably. During nectochaete development, most of the Hox genes participate in the formation of the body of the segmented larva, functioning in accordance with the rule of spatial colinearity, as in embryogenesis of other bilaterian animals. On the contrary, during postlarval development, Hox genes are expressed in a gradient manner in the worm’s body with morphologically similar segments. We suggested that the gradient expression of Hox genes in postlarval ontogenesis does not lead to the morphological diversity of segments, but creates the positional information, which is needed to determine the position of body parts in a homonomously segmented body (Bakalenko, Novikova and Kulakova, unpublished data).

Our data from this study support the hypothesis of a coordinating function of Hox genes. Indeed, for some of the genes we observed a rapid reorganization of expression patterns in differentiated cells long before the morphological signs of regeneration were visible. The patterns of most of the genes from the early and the middle response group are reorganized in such a way that the last segments of the operated worm acquire the Hox expression pattern characteristic of the posterior body end.

Normal Hox gene activity in *A. virens* lacks temporal colinearity, but the anterior expression boundaries are colinear. The anterior boundaries of the genes that pattern the larval body are stabilized in larval segments. The middle genes (*Nvi-Hox7*, *Nvi-Lox2* and *Nvi-Lox4*) are turned on after metamorphosis and have diffuse anterior boundaries, which are established late in the postlarval body (Bakalenko, Novikova and Kulakova, unpublished data). Interestingly, during pattern reorganization, the anterior boundaries of the genes that pattern the body of nectochaete larva, except for *Nvi-Post2*, remain stable. Oppositely, the expression boundaries of the genes that are turned on in the postlarval body can shift widely, but never spread to the larval segments. This interesting feature obviously reflects the difference in the epigenetic regulation of Hox transcription in larval and postlarval segments. In the segments where middle genes have been expressed at some moment (postlarval segments), their expression is easily induced, but in larval segments these genes have never been active and so cannot be turned on. It is interesting to note that the order of activation/repression of different Hox genes in the amputation site is not consistent with their presumed genomic order (Figure 13a). Here we observe that the greater the difference between the gene expression level at the prospective amputation site and that in terminal structures (pygidium and GZ) during normal development, the faster up- or downregulation of the gene expression proceeds during regeneration. Indeed, all the early response genes (*Nvi-Post2*, *Nvi-Lox2* and *Nvi-Lox5*) display high expression levels in terminal structures and low expression levels at the potential amputation site during normal development. Oppositely, the late response genes (*Nvi-Hox1*, *Nvi-Hox4*, *Nvi-Lox4*) display low expression levels both at the potential amputation site and in the GZ.

### Hox genes in regeneration of bilaterian animals

Hox gene expression during regeneration was studied in many representatives of Bilateria [4,19,20,22]. Nevertheless, only one work describes the expression pattern of almost the whole set of Hox genes (with the exception of *Lox4* and *Hox7*) during the polychaete regeneration [29]. The authors studied the expression of Hox genes in regeneration of a homonomously segmented polychaete *P. dumerilii*. Unfortunately, it is difficult to compare our results with those from that study. Pfeifer and colleagues analyze the gene expression at a stage when the blastema has already formed (1 dpa), active formation of terminal structures is in progress, and differentiation of tissues and organogenesis has started. During these stages of regeneration, the expression pattern of many Pdu-Hox genes is similar to the expression pattern of Nvi-Hox genes. The only difference is the expression of *Nvi-Lox2* and *Pdu-Lox2*. *Nvi-Lox2* is the expressed in the segmental ectoderm and the neural system, whereas the domain of *Pdu-Lox2* expression is located in coelothelia. However, the authors miss the early expression phase of some genes in differentiated tissues. This very phase is the reorganization of the expression pattern according to the new body proportions. Pfeifer and colleagues consider the expression of the Hox gene cluster in regenerating structures of *P. dumerilii* to pattern the neural system of nascent segments [29].

The expression of some Hox genes during regeneration was shown for another representative of Lophotrochozoa, planaria *Dugesia japonica*[20,21]. Notably, *DjAbd-Ba*, *Plox4-Dj* and *Plox5-Dj* genes, which display a gradient expression pattern in normal development, show reorganization of expression patterns in definitive tissues during regeneration similar to *A. virens*.

Among vertebrates (Deuterostomia), the best capacities for axial regeneration are characteristics of Urodela [3,16]. Surprisingly, these animals do display the persisting expression of Hox genes in adult tissues, which is upregulated during the regeneration processes [30,31]. Upregulation of 5′HoxC genes after tail excision was found in the neural system of adult newt *Pleurodeles waltl*. The authors consider that such a persisting expression of Hox genes in the neural system of an adult animal provides the positional information necessary for regeneration [30].

In earlier investigations, the expression of some Hox genes was found in the limbs and the tail of adult Urodela [31,32]. The most intriguing fact is that their orthologs are not expressed in the limb of *X. laevis*, whose adults cannot regenerate limbs [31]. The ability of tailed amphibians to regenerate is probably associated with the maintenance of Hox gene expression in definitive tissues [30 – 32].

In higher vertebrates, Hox gene transcripts were found in populations of stem cells, such as fibroblasts and mesenchymal stem cells, and in tissues where constant renewal is possible [9,10,23,24]. Mammals are incapable of epimorphic regeneration, and their wound healing is followed by scar formation, after which regeneration is impossible [33]. Nevertheless, mammals can regenerate internal organs because of activity of stem cells present in certain types of tissue [11,12]. Fibroblasts and mesenchymal stem cells were shown to carry the Hox code, which they get during embryogenesis [34,35]. Once established, this Hox code in stem cells is retained throughout the lifetime and cannot be changed in case of positional failure.

Thus, the mechanism of maintaining and restoring positional values seems to have a similar basis among representatives of different bilaterian lineages. The important element of this complex system is the genes of the Hox cluster, which are thought to be capable of maintaining positional information in adult tissues and are consistently expressed in response to injuries to restore the positional coordinates in a regenerating animal. We suggest that this capacity of Hox genes is expressed in different ways in different taxa. In mammals, the rigidity of positional memory, necessary for the correct differentiation of fibroblasts and mesenchymal stem cells in the context of their background, provides a well-adjusted physiological and organ regeneration at the expense of epimorphic regeneration. A large variety of tissues and the necessity to maintain tissue homeostasis and integrity in mammals call for the early commitment of the Hox code. The other strategy is used by animals capable of epimorphic regeneration: tailed amphibians, planarians and polychaetes. These animals maintain the positional information in the adult body because of Hox gene expression in differentiated tissues and can rapidly reorganize these expression patterns when the body proportions change.

### Regeneration of nereid polychaetes: regulation in more detail

When considering the early stages of regeneration of *A. virens* in more detail, we can see that immediately after the operation, the edges of the entodermal gut contact with the edges of the covering epithelium. During normal growth, the boundary between the ectodermal and the endodermal structures is mediated by the terminal structure, the pygidium. The narrow GZ, consisting of cells capable of rare synchronic divisions, lies between the ectoderm and the pygidium [36]. Based on well-known experimental works on regeneration of nereid polychaetes (reviewed in [37]) and other model organisms [16,38,39], we propose that the contact of the tissues on the border between the gut and the segmental ectoderm, which never come in contact during normal development, leads to the formation of an organizer, the source of morphogenic signals. These signals, addressed to the adjacent tissues, can initiate the reorganization of positional information, dedifferentiation, proliferation and formation of a new pygidium and GZ. We observe the first expression answer to these signals from the wound surface by 3–4 hpa in case of Nvi-Hox genes, which are expressed in the GZ and the pygidium during normal development (Figure 1c,k; Figure 2c,k, Figure 3c,k), as well as for *Nvi-Cad*[40]. *Nvi-Cad* belongs to the ParaHox gene group, which is known to be upstream regulators for Hox genes [41,42]. This may suggest a role for *Nvi-Cad* as a positional marker, whose well-timed changes in the expression boundaries lead to reorganization of spatial coordinates in the regenerating worm (Figure 13b).

All Hox genes of early response are activated in the nervous system. As it happens before the blastema formation, one can suggest the existence of a link between these two processes. Indeed, the blastema starts to form after the expression domains of the Hox genes have been reorganized according to the new body proportions. However, in a series of studies on nereid polychaete *Nereis diversicolor* performed in the 1970s (reviewed in [37]), it was shown that the restoration of the pygidium, the GZ and new segments happened in the area of contact between the gut and body wall independently of the neural system. According to the authors’ description, without the nervous system, the pygidium did not produce the anal cirri, and the nascent segments lacked the “cephalo-caudal differentiation” [37]. The early reorganization of Hox patterns probably does not define the blastema formation. Nevertheless, Hox genes may prepattern the blastema itself and its sources so that the new structures (pygidium, GZ and new segments) are integrated into the whole body correctly. Experiments on functional knockout of one or several genes of early response might provide interesting results, helping to reveal the regulatory connections between the Hox genes and to show the influence of Hox expression failure on segmentation and segmental differentiation.

### Expression of antisense transcripts

Rapid reorganization of Hox expression patterns during regeneration of *A. virens* demands well-coordinated regulation of this process. While studying Hox gene expression in the postlarval development of *A. virens*, expression of long non-coding RNAs, complementary to sense RNA probes of Hox genes, was shown for the first time for a lophotrochozoan animal (Bakalenko, Novikova and Kulakova, unpublished data). Long non-coding RNA transcripts are the transcripts that lack long open reading frames and do not code the proteins [43]. Discovered transcripts may belong to natural antisense transcripts (NAT) as they are at least partially complementary to known sequences of Hox genes. We found that antisense ncRNAs display unique expression patterns during normal growth of the worm (Bakalenko, Novikova and Kulakova, unpublished data). In this work we analyzed the expression dynamics of ncRNA for *Nvi-Нох5* and *Nvi-Нох7* during worm regeneration.

Expression of *Nvi-antiНох5* is upregulated in the ganglion of the last body segment by 4 hpa (Figure 11c, k). At this point, expression of the sense transcript hasn’t been reorganized yet. But in several hours, *Nvi-Hox5* expression moves towards the head, and *Nvi-antiНох5* expression intensifies on this territory. By 18–24 hpa, transcript patterns overlap in 1–2 ganglia in the middle of the body, and after 48 hpa transcripts are expressed in complementary domains. Thus, we can assume we observe the negative regulation of the sense transcript by the antisense one.

In the case of *Nvi-Нох7*, the different pattern of sense and antisense transcript overlapping is observed. These transcripts are expressed in the same ganglia of the nerve cord nearly at all regeneration stages, though the expression of *Nvi-antiНох7* is much less intensive (see “Results”).

Based on the complementary expression of *Nvi-Hox5* and *Nvi-antiHox5* transcripts, we can suggest that preceding activation of the antisense transcript provides very rapid negative regulation of the coding transcript by the non-coding one. The similar expression pattern and dynamics were shown for *Ubx* and *antiUbx* transcripts during the development of several myriapoda species: the activation of antisense *Ubx* occurred a bit earlier than *Ubx* expression, and transcripts displayed complementary expression patterns [44,45]. The authors proposed some models of negative regulation of the sense *Ubx* transcript by the antisense one [46-48], but all of these mechanisms of regulation are implemented in the cell nucleus. However, in our case and in the study on myriapoda, long ncRNA transcripts were observed in the cytoplasm, where they were detected by *in situ* hybridization. It is unclear why ncRNAs leave the nucleus and go to the cytoplasm and what mechanism may be activated to perform potential regulation of sense transcripts by antisense ones. Moreover, overlapping of sense and antisense expression domains was shown for both *Nvi-Hox5* and *Nvi-Hox7* genes on some regeneration stages. We had no opportunity to describe the distribution of Hox proteins at that time point. The suppression of gene expression may occur here on the translational level because of RNAi or another, still unknown mechanism.

## Conclusions

We showed that gradient Hox gene expression patterns undergo a rapid and consistent reorganization during the earliest stages of regeneration. We consider these changes to be orchestrated to compensate for the positional failure as they restore the native expression pattern in the rest of the body long before the first morphological signs of regeneration. The expression dynamics of Hox genes in a regenerating polychaete *A. virens* shows that the establishment, maintenance and restoration of positional memory in a multisegmental worm may be mediated by this group of genes.

Antisense transcripts of Nvi-Hox genes are expressed in spatial domains partially complementary to protein-encoding RNA. Their expression dynamics during regeneration indicate repression rather than activation of complementary mRNA. Regulatory transcripts are localized in cytoplasm, which implies their participation in translational silencing, probably with the use of the mechanism of RNA interference.

Comparing the definitive expression of Hox genes in representatives of different evolutionary branches, we can suppose that the common ancestor of Bilateria already possessed the system of establishing and maintaining the positional information in the adult body using the Hox genes. This Hox genes’ ability was utilized in different ways in various animal taxa. Thus, mammals used the system of positional markers for establishing different types of differentiation of multipotent stem cells along the body axis and in this way acquired the ability to maintain tissue homeostasis. Tailed amphibians, planarians and polychaetes use the same principle of positional marking of cell territories by means of Hox genes, but they have become capable of (or retained the ability to) rapid reorganization of this information in the case of positional failure, thus providing themselves with outstanding regeneration capacities.

## Abbreviations

hpa: Hour post amputation; dpa: Day post amputation; GZ: Growth zone; VNC: Ventral neural cord.

## Competing interests

The authors declare that they have no competing interests.

## Authors’ contributions

ELN, NIB and MAK conceived the study, performed the experiments and the data analysis, and drafted the manuscript. AYN participated in material collection, maintaining of the worm culture and editing of the manuscript. All the authors have read and approved the final manuscript.
