# Correction: Expression of Hox genes during regeneration of nereid polychaete *Alitta virens* (Annelida, Lophotrochozoa)

**DOI:** 10.1186/2041-9139-4-21

**Published:** 2013-08-06

**Authors:** Elena L Novikova, Nadezhda I Bakalenko, Alexander Y Nesterenko, Milana A Kulakova

**Affiliations:** 1 Saint-Petersburg State University, department of embryology, laboratory of experimental embryology, Petergof Oranienbaumskoe sh., 2, Saint Petersburg, Russia

## 

After publication it was brought to our attention that there is an ambiguity in the title and in the abstract of our paper [1] that could lead to incorrect understanding of the name of the studied species. To avoid this, the correct title should read “Expression of Hox genes during regeneration of nereid polychaete *Alitta virens* (Annelida, Lophotrochozoa)” and the sentence “We have recently described the expression of 10 out of 11 Hox genes during postlarval growth of *Alitta (Nereis) virens*” in the abstract should read as “We have recently described the expression of 10 out of 11 Hox genes during postlarval growth of *Alitta virens* (formerly *Nereis virens*).”
